# Supplementary material for: Prediction of Potential Suitable Distribution Areas for an Endangered Salamander in China
Source: Animals (Basel). 2024 May 6;14(9):1390. doi: 10.3390/ani14091390 (PMC11083405; doi:10.3390/ani14091390)
Supplement: Supplementary file 1 [file animals-14-01390-s001.zip › animals-2954587-SI.pdf]

Table S1. The distribution data of *Liua shihi* collected in published literatures.

Species	Longitude	Latitude	Reference
Liua shihi	106.4036	29.8116	Li, H.; Liu, X. Investigation on amphibian and reptile resources in Jinyun Mountain Nature Reserve of Chongqing City. *Guizhou Agricultural Sciences* **2010**, *38*, 170-172. (In Chinese with English Abstract)
Liua shihi	107.6	31.5	Zeng, X.M.; Fu, J.Z.; Chen, L.Q.; etc. Cryptic species and systematics of the hynobiid salamanders of the Liua-Pseudohynobius complex: Molecular and phylogenetic perspectives. Biochem Syst Ecol 2006, 34, 467-477, doi:10.1016/j.bse.2006.01.006.
Liua shihi	108.45	31.52	
Liua shihi	108.58	31.78	
Liua shihi	109.91	31.33	
Liua shihi	110.14	31.17	
Liua shihi	110.25	31.41	
Liua shihi	110.27	31.43	
Liua shihi	110.28	31.43	
Liua shihi	109.8	31.48	
Liua shihi	108.6716	31.9534	Luo, J.; Gao, H.; Wang, Y.; etc. Study and Protection of Amphibian Species Diversity in Chongqing. Sichuan Journal of Zoology 2005, 378-385. (In Chinese with English Abstract)
Liua shihi	109.4705	31.0246	
Liua shihi	109.5764	31.4049	
Liua shihi	110.3476	31.0481	
Liua shihi	110.6825	31.7505	
Liua shihi	110.9844	30.8319	
Liua shihi	108.9425	30.2972	Li, S.B.; Su, Z.L.; Tong, P.; etc. Investigation on amphibian and reptile resources in Xingdoushan National Nature Reserve of Hubei Province. Sichuan Journal of Zoology 2010, 29, 130-133. (In Chinese with English Abstract)
Liua shihi	109.1466	29.6711	
Liua shihi	109.4866	30.301	
Liua shihi	108.0415	32.0874	Su, Y.; Luo, J.; Gan, X.P.; etc. Investigation and Evaluation of Amphibians in Hua 'eshan National Nature Reserve. *Journal of Southwest China Normal University(Natural Science Edition)* **2016**, *41*, 92-98. (In Chinese with English Abstract)
Liua shihi	109.8855	31.0805	Tian, W.S.Z.M. New species of amphibians published by Professor Liu Chengzhao during his lifetime. Sichuan

			Journal of Zoology 2000, 105-107. (In Chinese)
Liua shihi	109.9028	31.33713333	Zhang, P.; Zeng, X.; Fu, J.; etc. UCE Phylogenomics, detection of a putative hybrid population, and one older mitogenomic node age of Batrachuperus salamanders. Mol Phylogenet Evol 2021, 163, doi:10.1016/j.ympev.2021.107239.
Liua shihi	110.7475	32.0617	Liu, H.; Ai, H.; Ren, S.; etc. Investigation on the diversity of amphibians and reptiles in Yerengu Nature Reserve, Fangxian County, Hubei Province. *Sichuan Journal of Zoology* **2010**, *29*, 560-564. (In Chinese with English Abstract)
Liua shihi	110.7534	31.3545	Shu, S.; Dai, Z.; Wu, F.; etc. Investigation on resources of amphibians and reptiles in Wanchaoshan Nature Reserve of Hubei Province. *Journal of Central China Normal University (Natural Sciences)* **2007**, 278-281. (In Chinese with English Abstract)
Liua shihi	111.0526	30.1712	Dai, Z.; Zheng, Z.; Gong, R.; etc. Investigation on amphibian and reptile resources in Houhe National Nature Reserve of Hubei Province. *Chinese Journal of Zoology* **2009**, *44*, 48-53. (In Chinese with English Abstract)
Liua shihi	111.2135	30.4786	Tian, K.; Wang, Z.X.; Lei, Y.; etc. Investigation on the Biodiversities of Amphibians and Reptiles in Bengjianzi Nature Reserve，Hubei Province. Sichuan Journal of Zoology 2016, 35, 452-458. (In Chinese with English Abstract)
Liua shihi	111.2674	31.8838	Zeng, L.; Shu, S.; Wang, Q.; etc. Preliminary study on amphibians and reptiles in Wudaoxia Nature Reserve of Hubei Province. *Journal of Hubei University(Natural Science)* **2008**, *30*, 407-410. (In Chinese with English Abstract)
Liua shihi	113.4345	32.3851	Zhao, H.; Wang, Q.; Wang, C.; etc. Status and Fauna Analysis of Amphibian Resources in Henan Province. *Journal of Henan University(Natural Science)* **2015**, *45*, 705-711. (In Chinese with English Abstract)
Liua shihi	115.4135	31.8043	Wang, X.; Pei, X.; Xu, Y.; etc. Investigation of amphibian diversity in Huangbai Mountain National Forest Park of Henan Province. *Chinese Journal of Zoology* **2011**, *46*, 53-56. (In Chinese with English Abstract)

Table S2. The distribution data of *Liua shihi* collected on website.

Species	Longitude	Latitude	Sourse
Liua shihi	107.996238	32.822187	The Global Biodiversity Information Facility website (http://www.gbif.org)
Liua shihi	108.347116	30.945958	
Liua shihi	108.85	30.3	
Liua shihi	109.08747	31.77473	
Liua shihi	109.848823	31.485095	
Liua shihi	109.881853	31.071815	
Liua shihi	109.897798	31.33769	
Liua shihi	109.91026	31.37432	
Liua shihi	109.969182	31.406233	
Liua shihi	110.09431	31.361518	
Liua shihi	110.131327	31.454329	
Liua shihi	110.266667	31.216667	
Liua shihi	110.310421	31.435606	
Liua shihi	110.392936	31.020833	
Liua shihi	110.420354	31.456274	
Liua shihi	110.45575	31.40684	
Liua shihi	110.619154	31.656546	
Liua shihi	110.680447	31.743483	
Liua shihi	111.04204	31.01894	
Liua shihi	112	31	
Liua shihi	109.3685	32.3948	

Table S3. The distribution data of *Liua shihi* collected from field survey.

Species	Longitude	Latitude	The field survey site
Liua shihi	109.9823512	31.41700603	Wushan County, Chongqing City
Liua shihi	109.9819247	31.41689044	
Liua shihi	109.9825162	31.41703236	
Liua shihi	109.9826409	31.41705296	
Liua shihi	109.8856938	31.36656954	
Liua shihi	109.9815334	31.47246185	
Liua shihi	109.8855919	31.36653748	
Liua shihi	109.8854926	31.36650083	
Liua shihi	109.1898818	31.52490621	Wuxi County, Chongqing City
Liua shihi	109.1901729	31.5247736	
Liua shihi	109.1901018	31.52477246	
Liua shihi	109.1902064	31.52454725	
Liua shihi	109.1747716	31.49160188	
Liua shihi	108.9422603	31.63721609	
Liua shihi	108.9423877	31.63763512	
Liua shihi	108.9418392	31.63630721	
Liua shihi	108.9419062	31.63666003	
Liua shihi	108.9420685	31.6372001	
Liua shihi	108.9421396	31.63708249	
Liua shihi	108.9421463	31.63742618	
Liua shihi	108.9422026	31.63737251	
Liua shihi	108.9423877	31.6377493	
Liua shihi	108.9423139	31.63782009	
Liua shihi	108.9416592	31.63675978	
Liua shihi	108.9424789	31.63796396	
Liua shihi	108.9428665	31.63804617	
Liua shihi	109.0714288	31.80306241	Chengkou County, Chongqing City
Liua shihi	108.9976318	31.80571915	
Liua shihi	108.8901074	31.78364575	
Liua shihi	108.5450077	31.88457261	
Liua shihi	108.5449541	31.88450884	
Liua shihi	108.5448857	31.88441091	
Liua shihi	108.5448374	31.88433006	
Liua shihi	108.544765	31.88421846	
Liua shihi	109.06666	31.81666	
Liua shihi	108.6573494	32.06806518	
Liua shihi	110.4102339	31.41094468	Shennongjia Forestry District, Hubei Province
Liua shihi	110.4072545	31.42312594	
Liua shihi	110.3820799	31.4353101	
